# *In Vivo* Validation of Predicted and Conserved T Cell Epitopes in a Swine Influenza Model

**DOI:** 10.1371/journal.pone.0159237

**Published:** 2016-07-13

**Authors:** Andres H. Gutiérrez, Crystal Loving, Leonard Moise, Frances E. Terry, Susan L. Brockmeier, Holly R. Hughes, William D. Martin, Anne S. De Groot

**Affiliations:** 1 Institute for Immunology and Informatics, Department of Cell and Molecular Biology, University of Rhode Island, Providence, RI, United States of America; 2 Virus and Prion Diseases Research Unit, NADC, USDA ARS, Ames, IA, United States of America; 3 EpiVax Inc., Providence, RI, United States of America; Icahn School of Medicine at Mount Sinai, UNITED STATES

## Abstract

Swine influenza is a highly contagious respiratory viral infection in pigs that is responsible for significant financial losses to pig farmers annually. Current measures to protect herds from infection include: inactivated whole-virus vaccines, subunit vaccines, and alpha replicon-based vaccines. As is true for influenza vaccines for humans, these strategies do not provide broad protection against the diverse strains of influenza A virus (IAV) currently circulating in U.S. swine. Improved approaches to developing swine influenza vaccines are needed. Here, we used immunoinformatics tools to identify class I and II T cell epitopes highly conserved in seven representative strains of IAV in U.S. swine and predicted to bind to Swine Leukocyte Antigen (SLA) alleles prevalent in commercial swine. Epitope-specific interferon-gamma (IFNγ) recall responses to pooled peptides and whole virus were detected in pigs immunized with multi-epitope plasmid DNA vaccines encoding strings of class I and II putative epitopes. In a retrospective analysis of the IFNγ responses to individual peptides compared to predictions specific to the SLA alleles of cohort pigs, we evaluated the predictive performance of PigMatrix and demonstrated its ability to distinguish non-immunogenic from immunogenic peptides and to identify promiscuous class II epitopes. Overall, this study confirms the capacity of PigMatrix to predict immunogenic T cell epitopes and demonstrate its potential for use in the design of epitope-driven vaccines for swine. Additional studies that match the SLA haplotype of animals with the study epitopes will be required to evaluate the degree of immune protection conferred by epitope-driven DNA vaccines in pigs.

## Introduction

Swine influenza is a highly contagious respiratory viral infection in pigs that has a major impact on their health. In addition, influenza outbreaks are responsible for significant financial losses to pig farmers, large and small, on an annual basis [1]. The negative economic impact is due to weight loss, reduced weight gain and predisposition to other infections [2]. Clinical signs of the disease include fever, coughing, sneezing, nasal discharge, lethargy, and anorexia. The causative agent is influenza A virus (IAV), a negative-sense, single-stranded, segmented RNA virus of the *Orthomyxoviridae* family. Transmission is by direct contact and by aerosol [3]. As is true with IAV in humans, antigenic drift by accumulation of mutations and/or antigenic shift by reassortment with genes from other IAV subtypes results in the emergence of novel influenza viruses [4]. Human-to-swine ‘spillover’ events also contribute to the genetic diversity of swine IAV [5]. H1N1, H1N2, and H3N2 swine IAV subtypes are endemic and co-circulate in swine in the U.S. [6].

Continual reassortment events led to the emergence of a novel triple-reassortant internal gene (TRIG) cassette that contains internal genes derived from human (PB1 gene), avian (PA and PB2 genes) and swine (NS, NP, and M genes) IAV viruses [7]. The TRIG is conserved among swine IAV circulating subtypes and it seems to have the ability to combine with numerous hemagglutinin (HA) and neuraminidase (NA) genes, including those of human and swine origin leading to enhanced strain variability [7]. Thus, the primary antigenic component of swine IAV vaccines is HA, which has evolved to present antigenically distinct HA lineages including: (1) the classical swine lineages, H1α, H1β, H1γ, H1γ-2; (2) lineages derived from human seasonal H1 viruses, H1δ1, H1δ2; the H1pdm09; and (3) H3 cluster I-IV viruses [6,8,9]. This marked genetic diversity complicates the development of effective vaccines for pigs.

The predominant type of vaccine used by pork producers consists of whole inactivated viruses (WIV), administered with adjuvant by intramuscular injection. HA is the primary target of protective antibody responses of this platform. These vaccines are problematic for three reasons. First, antibody induced by WIV vaccination does not provide significant protection against antigenically diverse strains of IAV [8,10]. Second, WIV vaccines have been linked to vaccine-associated enhanced respiratory disease (VAERD) in pigs when WIV vaccine and infecting strains are mismatched [11–13]. Lastly, existing vaccines do not adequately address viral diversity.

In contrast, cell-mediated immune responses to epitopes that are conserved across IAV strains have been shown, in a number of studies, to be protective against influenza. For example, human and mouse studies demonstrate that cell-mediated responses to conserved non-structural proteins can be broadly cross-reactive [14] and protective against variety of IAV subtypes [15]. Both CD4+ T helper cells (Th) [16] and CD8+ cytotoxic T cells (CTL) [17,18] contribute to clearance of IAV. T cell help is also required for the development of high titers of strain-specific antibody [19]. In fact, memory T cell response improves vaccine efficacy against emerging IAV strains when cross-reactive helper T cell populations are present from prior infection and/or vaccination [20,21]. CTL responses have also been associated with viral clearance and reduced clinical severity in mice and humans [22,23]. Our group has been interested in the role of cross-conserved epitopes in protection against IAV in human populations, and has postulated that immunity to cross-conserved epitopes may have contributed to attenuation of morbidity in some age groups during the 2009 H1N1 IAV pandemic [24].

Adaptive cell-mediated immune response depends on T cell receptor (TCR) recognition of peptides bound to major histocompatibility complex (MHC) molecules presented on the surface of cells. Immunoinformatics tools have accelerated the discovery of T cell epitope peptides and design of epitope-driven vaccines (EDV) for human IAV [25–28]. The lack of quantitative MHC binding data has limited the development of tools for swine, cattle, and other food animal species. We recently developed a new tool for swine epitope prediction (PigMatrix) that leverages the pocket profile method originally described by Sturniolo et al. [29]. We integrated the new swine MHC predictions into iVAX, the suite of tools for vaccine design that were validated in a number of pre-clinical studies of human vaccines [30,31]. This set of tools is particularly useful for identifying T cell epitopes that are conserved across subtypes of strains [32], which is relevant to develop a IAV vaccine for pigs. Having integrated the new matrices into this ‘*in silico* vaccine design’ platform, we were able to apply the PigMatrix version of iVAX to IAV.

In this study, we used PigMatrix to predict class I and II T cell epitopes that are conserved in external and internal proteins from seven circulating IAV strains. We selected epitopes predicted to bind to SLA alleles that were *previously reported* to be prevalent in outbred U.S. swine populations [33,34] and developed a prototype PigMatrix epitope-driven DNA-vaccine (PigMatrix-EDV) as a tool to evaluate immunogenic responses to highly conserved predicted epitopes in a swine IAV model. PigMatrix predicted peptides induced specific interferon gamma (IFNγ) recall responses in pigs immunized with the prototype PigMatrix-EDV encoding strings of class I and II putative epitopes. In addition, we performed a retrospective analysis to compare IFNγ responses to individual peptides (28 class I and 20 class II peptides) with predictions specific to the SLA expressed in the study cohort. The results showed that cohort-specific predictions using PigMatrix, were particularly effective for identification of non-immunogenic peptides.

## Material and Methods

### Sequences

Gene sequences of proteins expressed by seven representative swine IAV (pandemic A/California/04/2009 (H1N1) (H1N1pdm09), A/swine/Illinois/5265/2010 (H1N1) (IL/10), A/swine/Ohio/511445/2007 (H1N1) (OH/07), A/swine/Minnesota/02011/2008 (H1N2) (MN/08), A/swine/Minnesota/A01301731/2012 (H1N2) (MN/12), A/swine/Texas/4199-2/1998 (H3N2) (TX/98), A/turkey/Ohio/313053/2004 (H3N2) (OH/04)) [35,36] were downloaded from the Influenza Virus Resource [37] (S1 Table).

### Conservation analysis

The goal of the conservation analysis was to identify highly cross-conserved 9-mer peptides. Since 9-mers fit into the SLA binding groove [38], proteins derived from IAV genomes were parsed into 9-mer frames overlapping by eight amino acids using the Conservatrix algorithm [32]. Nine-mer sequences were searched for identically matched segments among IAV strains, as previously described [30]. Resulting 9-mers were ranked by their conservation within the dataset.

### T cell epitope prediction

Using the pocket profile method [29] and well-defined EpiMatrix binding preferences for human MHC pockets, we developed PigMatrix prediction matrices as previously described [39]. Matrices were designed based on the binding preferences of the best-matched Human Leukocyte Antigen (HLA) pocket for each SLA pocket. The contact residues involved in the binding pockets were defined from crystal structures of SLA or HLA supertype alleles for class I and II, respectively. Allele selection was based on prior data indicating their prevalence in outbred swine populations [33,34]. Matrices were constructed to predict T cell epitope binding to class I (SLA-1*0101, 1*0401, 2*0101, and 2*0401) and class II (SLA-DRB1*0101, 0201, 0401, and 0601) SLA alleles. SLA-1*0401, 2*0401 and SLA-DRB1*0201 were previously validated using published epitopes [39]. We also developed matrices for SLA alleles expressed in the study cohort (cohort-specific prediction) to perform a retrospective analysis.

All highly conserved 9-mers resulting from Conservatrix analysis were scored for binding potential against the panel of SLA alleles. PigMatrix raw scores were standardized to Z-scores to compare potential epitopes across multiple SLA alleles. Peptides with Z-scores above 1.64 (the top 5% of any given sample of 9-mers) were identified as likely to be SLA ligands. The final selection of putative SLA class I-restricted epitopes was based on PigMatrix score (Z-score>1.64), SLA class I allele coverage (≥50%) and IAV strain coverage.

### Construction of immunogenic consensus sequences

EpiAssembler was used to construct 16–25 amino acid length SLA-DRB1-restricted sequences that were highly conserved in IAV strains, promiscuous (predicted to bind to multiple alleles), and enriched for immunogenicity (immunogenic consensus sequences or ICS) [30]. The density of predicted binding motifs in each ICS was scored (i.e. cluster score) using ClustiMer [30]. The cluster score represents the deviation in predicted epitope content from baseline expectation based on random peptides [40]. ICS with cluster scores above 10 were considered to be high-quality clusters for inclusion in the prototype vaccine. Peptides were ranked based on cluster score and IAV strain coverage and the final selection of epitopes was made using the same three criteria described above for class I peptides. Highly hydrophobic peptides were excluded as these are known to be more technically difficult to synthesize and may be less soluble in aqueous solutions.

### Multi-epitope plasmid DNA vaccine engineering and production

Predicted epitope sequences were concatenated to form two multi-epitope genes (one for SLA class I and one for class II epitopes). VaccineCAD [41] and a concatemer optimization algorithm (unpublished) were used to rearrange the peptides to avoid creation of novel epitopes at peptide junctions and to search for transmembrane helices that might interfere with production of the epitope concatemer proteins. Both algorithms, VaccineCAD and the concatemer optimization algorithm, used PigMatrix to predict junctional epitopes.

Transmembrane helices were predicted using TMHMM 2.0 [42]. In addition, where reordering did not sufficiently reduce the potential for junctional immunogenicity, a cleavage promoting spacer (‘AAY’) for class I-restricted constructs [43] or a binding inhibiting ‘breaker’ sequence (‘GPGPG’) for class II-restricted constructs [44], was introduced between peptides to optimize epitope processing. Two genes (one for class I and one for class II epitopes) predicted to have no transmembrane segments or junctional epitopes, were codon-optimized and synthesized by GeneArt (Life Technologies, NY, USA). Tandem stop codons were incorporated downstream of the epitope sequences. Class I and class II genes, respectively, were subcloned at predefined flanking restriction sites downstream of either a destabilizing UbiquitinA76 tag (UbA76) in pNTC8684-eRNA41H for proteasome targeting and a tissue plasminogen activator (TPA) leader sequence in pNTC8682-eRNA41H (Nature Technology Corporation, NE, USA) for secretory pathway targeting. High-purity plasmids for immunizations were prepared by Nature Technology Corporation, Inc. at research grade. Each plasmid underwent quality control testing including spectrophotometric concentration and A_260_/A_280_ ratio determination (1.97), restriction digest analysis to assure the presence of the multi-epitope genes, agarose gel electrophoresis determination of residual host RNA and DNA (none detected), and quantitative endotoxin testing (<2.0 EU/mg).

### Peptide synthesis

Peptides corresponding to putative epitopes in the DNA vaccine were synthesized using 9-fluoronylmethoxycarbonyl (Fmoc) chemistry by 21st Century Biochemicals (Marlboro, MA). Peptide purity was >80% as ascertained by analytical reversed phase HPLC. Peptide mass was confirmed by tandem mass spectrometry.

### Immunizations

**Fig 1:**
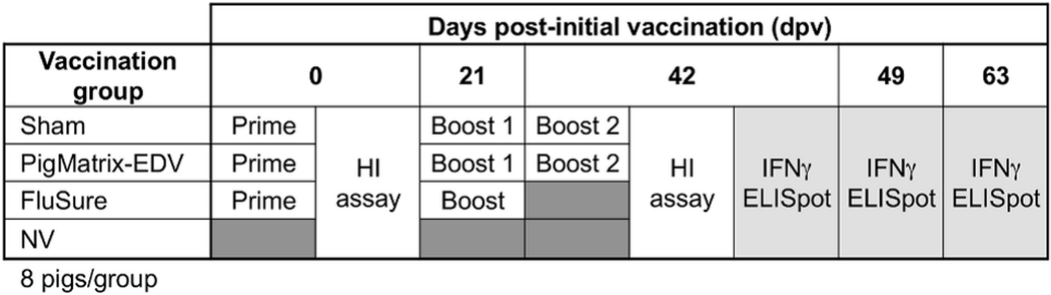
Experimental outline.

Thirty-two, 3-week old outbred pigs from a high-health status herd known to be free of IAV were delivered to the USDA-National Animal Disease Center. To ensure that prior exposure to IAV resulting in immunity was absent, all of the pigs were screened for influenza A nucleoprotein antibody by ELISA (MultiS ELISA, IDEXX, Westbrook, Maine) prior to the start of the study. All of the study pigs were treated with ceftiofur crystalline-free acid (Excede; Zoetis Animal Health, Florham Park, NJ, USA) and enrofloxacin (Baytril 100; Bayer HealthCare AG, Monheim, Germany) upon arrival to reduce bacterial contaminants. The experimental outline is summarized in Fig 1. Pigs were randomly distributed into four groups of eight and housed in separate isolation rooms in animal biosafety level 2 (ABSL2) containment. Three groups were vaccinated: (i) one group of eight pigs was vaccinated with the prototype PigMatrix DNA-vaccine as the initial prime vaccination, followed by two homologous boosts at 21 and 42 days post-initial vaccination (dpv) (PigMatrix-EDV); (ii) one group of eight pigs was vaccinated with empty DNA plasmids containing no epitopes (Sham); (iii) one group of eight pigs was vaccinated with commercially available FluSureXP^®^ administered 21 days apart, according to the manufacturer’s directions (Zoetis Animal Health, Florham Park, NJ) (FluSure). FluSureXP^®^ contains whole inactivated γ-cluster H1N1, δ1-cluster H1N1, δ2-cluster H1N1, and cluster IV H3N2 swine IAV viruses. The final group of eight non-vaccinated pigs served as controls (NV). The prototype PigMatrix-EDV plasmids were thawed at 4°C overnight, combined and administered intramuscularly in the postauricular region of the neck by needle stick injection with 4 mg per DNA plasmid in 4 mL of Tris-EDTA (TE) buffer (2 mL on right side and 2 mL on left side).

### Animal care

Animals at the National Animal Disease Center (NADC) are cared for in accordance with the guidelines set forth in the “Guide for the Care and Use of Laboratory Animals” (National Academy Press, 1996) and in regulations and standards as promulgated by the Agricultural Research Service, USDA, pursuant to the Laboratory Animal Welfare Act of August 24, 1966, as amended. Animal studies are reviewed and approved by NADC’s Institutional Animal Care and Use Committee (IACUC). In addition, the IACUC is federally mandated to review, at least once every 6 months, the research facility's animal care program and physical facilities per USDA regulations and using the “Guide for the Care and Use of Laboratory Animals” as the basis for review. Full-time animal caretakers, technicians and supervisors and on-call veterinarians perform routine animal care, as well as weekend/holiday activities and respond to emergencies. NADC staff members who worked with the animals have backgrounds and continuing training in the appropriate, species-specific care and handling of research animals. Training courses for animal staff include safe handling skills, animal welfare, specific procedures (e.g. bleeding), personal protective equipment (PPE) requirements, as well as proper handling and care and use of anesthetics and analgesics. For this study, animals were housed in an ABSL-2 facility (12 h light/dark cycle) during the course of the study, and humanely euthanized at the termination of the project with a lethal dose of pentobarbial (Fatal Plus; Vortech Pharmaceuticals, Dearborn, MI). Protocols were in place to humanely euthanize any animals if unforeseen clinical disease presented, such as severe lameness or depression that results in recumbency with reluctance to stand, although that did not occur in this study (all animals in the study were terminated at the end of the experiment). Animal observations and feedings were completed at least twice daily by personnel who have been trained to look for signs of illness or abnormalities, at which time the veterinarian on-call and the principal investigator would have been notified.

### Measurement of IFNγ response by ELISpot assay

At 42, 49 and 63 dpv, whole blood was collected by venipuncture and peripheral blood mononuclear cells (PBMC) were isolated as previously described [45]. The frequency of epitope-specific T cells was determined by porcine IFNγ enzyme-linked immunosorbent spot (IFNγ ELISpot) assay according to the manufacturer’s recommendations (R&D Systems, Minneapolis, MN). Wells were seeded with 2.5 x 10^5^ PBMCs and stimulated with pooled peptides at 10 μg/mL, whole H1N1pdm09 virus (WV) at a multiplicity of infection (MOI) of 0.5, pokeweed mitogen (PWM) at 1 μg/mL, or culture media in a final volume of 0.25 mL.

**Fig 2:**
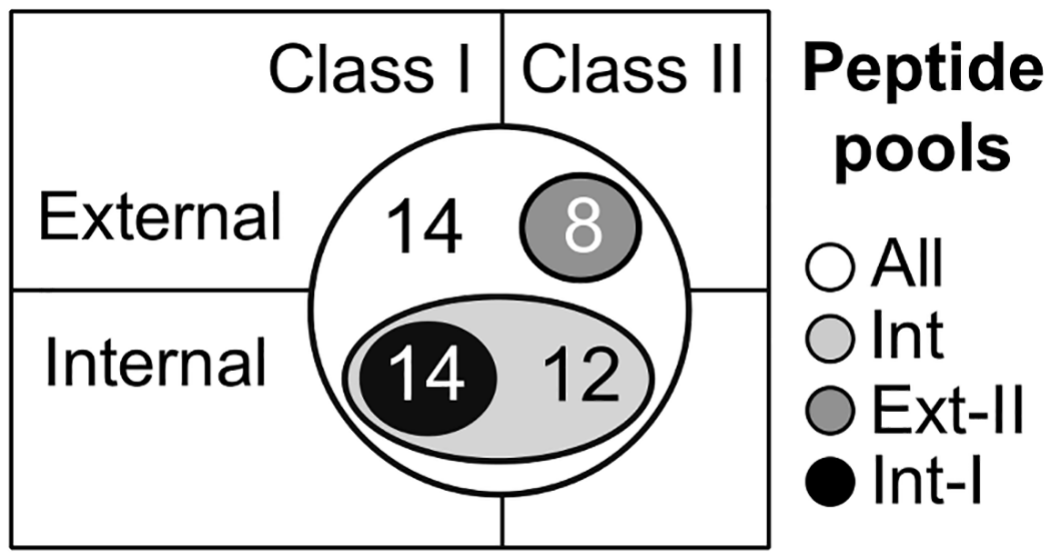
Peptide pools tested. All: 48 peptides. Int: 26 class I and II peptides predicted from internal proteins. Ext-II: Eight class II peptides predicted from external proteins. Int-I: 14 class I peptides predicted from internal proteins.

Immune responses to the IAV epitopes contained in the vaccines were evaluated using PBMC from each of 32 study animals. To simplify the analysis, four pools of peptides were evaluated at all PBMC sampling points—one that included all 48 predicted peptides (All); a second pool that contained 26 class I and II peptides predicted from internal proteins (Int); a third pool that contained 8 class II peptides predicted from external proteins (Ext-II); and a final pool that contained 14 class I peptides predicted from internal proteins (Int-I) (Fig 2).

In addition to the assays that were performed using pooled peptides, we evaluated epitope-specific IFNγ responses to individual peptides at 49 dpv, using PBMC from pigs in groups PigMatrix-EDV and FluSure (five from each group). Triplicate assays were performed for all peptide stimulations and for controls. After 18 h of incubation in a 37°C humidified 5% CO_2_ incubator, the ELISpot plates were washed and developed according to the manufacturer’s recommendations. The ELISpot plates were then scanned in a CTL-ImmunoSpot S5 UV analyzer and spot counts were recorded using the ImmunoSpot software (Cellular Technology Ltd., Shaker Heights, OH). Results were recorded as the average number of spot forming cells (SFC) over background and adjusted to spots per one 10^6^ PBMC. A response was considered positive if the number of spots was greater than or equal to 20 SFC over background per 10^6^ PBMC.

At the end of the study, pigs were SLA-typed using a low-resolution group-specific typing method [33] to evaluate SLA diversity and correlate epitope predictions with IFNγ responses. Select pigs were typed: two pigs from group NV, eight from Sham, seven from PigMatrix-EDV, seven from FluSure.

#### Retrospective analysis

IFNγ responses to individual peptides were compared to predictions using cohort-specific class I and II SLA PigMatrices. Class I peptides were scored and considered potential binders if the mean of significant Z-scores was above 1.64. Class II peptides with cluster scores above 10 were categorized as potential ligands. Experimentally, peptides that induced more than 20 SFC over background per 10^6^ PBMCs in at least one pig were considered positives. Based on the comparison of experimental results and predictions, peptides were divided into one of four categories (true positives, true negatives, false positives, and false negatives). True-positive peptides were predicted and validated *in vitro* as immunogenic, while true-negative peptides were predicted and biologically validated to be non-immunogenic. False negative peptides were predicted to be non-immunogenic, yet produced a positive response; false positive peptides were predicted to be immunogenic, but produced no response in the IFNγ ELISpot assay. To evaluate the predictive performance of the matrices, we calculated the positive and negative predictive values (PPV and NPV, respectively) and area under the receiver operating characteristic (ROC) curve (AUC) using the sensitivity and 1—specificity (false positive rate) values.

### Antibody evaluation

Pig serum was collected at 0 and 42 dpv for hemagglutination inhibition (HI) assay to assess antibody responses following vaccination as described previously [46]. Briefly, sera were heat-inactivated at 56°C for 30 min and then treated with a 20% suspension of kaolin (Sigma-Aldrich, St. Louis, MO) and subjected to adsorption with 0.5% turkey red blood cells (RBC) to remove nonspecific hemagglutinin inhibitors and natural serum agglutinins. The HI assays were then performed using H1N1pdm09 and OH/07 (γ-cluster H1) strains as antigen. Titers were determined using 2-fold serial dilutions to detect the reciprocal endpoint of HI, log_2_ transformed and reported as the average geometric mean reciprocal titer for each group. Sera with titers <40 were considered HI negative or suspect.

### Statistical analysis

IFNγ responses to restimulation treatments (pooled peptides and WV) in the PigMatrix-EDV group and the FluSure group, measured at 42, 49 and 63 dpv, were compared using a Kruskall-Wallis test followed by side by side comparisons of the groups using Dunn’s correction for multiple comparisons. The same test was used for comparison of HI antibody titers between groups at 42 dpv. Wilcoxon matched-pairs test was used to compare IFNγ responses within groups. To evaluate IFNγ responses to more than two restimulation treatments for a group at a specific timepoint and the effect of the boosts in the PigMatrix-EDV group, the Friedman test using Dunn’s correction was used. P values of less than 0.05 were considered significant. All the statistical analyses were performed using GraphPad Prism software (GraphPad, San Diego, CA).

## Results

### Epitope selection

**Fig 3:**
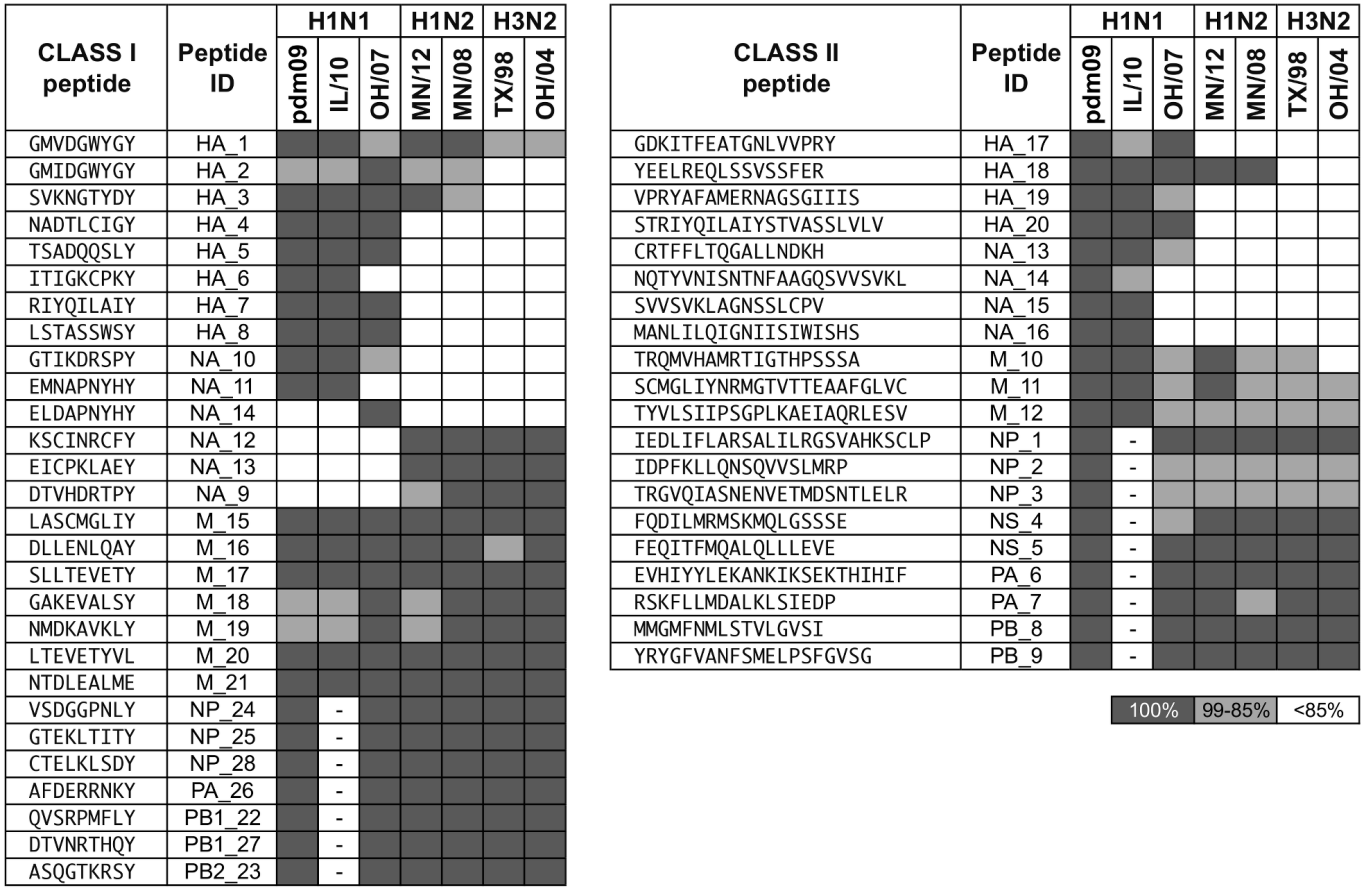
Class I and II predicted peptides. Peptides were selected based on predicted binding to class I and class II SLA alleles and conservation in IAV strains. The identity percentage between peptides and IAV strains is shown (100% dark gray, 99% - 85% gray, and <85% white). The Peptide ID is coded to the source protein. Sequences not available are marked with -.

A total of 28 class I and 20 class II peptides were down-selected for inclusion in the prototype PigMatrix-EDV IAV vaccine (Fig 3), following immunoinformatic predictions. Peptides were selected based on predicted binding to class I (SLA-1*0101, 1*0401, 2*0101, and 2*0401) and class II (SLA-DRB1*0101, 0201, 0401, and 0601) SLA alleles.

Since external proteins (HA and NA) are highly variable, it was difficult to identify highly conserved potential epitopes. For this study, the minimum IAV strain coverage required for epitopes derived from HA and NA proteins was 25%. We selected epitopes to achieve the broadest possible coverage despite this constraint. In contrast, internal proteins are conserved due to the presence of the TRIG cassette; therefore, the coverage threshold for peptides selected from internal proteins (M1, M2, NP, NS1, NS2, PA, PB1, PB1-F2, PB2) was 85%.

Fourteen class I peptides were selected from external proteins and 14 peptides were selected from internal proteins. The mean Z-score of class I peptides was 2.87(1.03), [reported as mean(standard deviation)]; these are high-scoring peptides that are considered likely to be T cell epitopes. Twenty-four of the class I peptides (85.7%) were predicted to bind to four alleles. Eleven of the 14 class I peptides (78.6%) identified in the external proteins were >85% identical in at least three of seven IAV strains. Similar epitopes were selected to evaluate strain specificity; HA_1 and HA_2 differed by one amino acid, but HA_1 was 100% identical in four strains, whereas HA_2 was present only in one IAV (OH/07). NA_14 was identified in one IAV, but its sequence was 77.8% identical (7 of 9 amino acids) to NA_11, which was conserved in two other IAVs. Both peptides were predicted to bind to four class I SLA alleles. For the putative class I peptides derived from internal proteins, 11 of 14 (78.6%) were 100% identical in the IAVs analyzed.

Cluster scores for all the selected class II ICS were greater than 10. Eight of the 20 ICS were derived from external and 12 from internal proteins. Their lengths ranged from 16 to 25 amino acids. All the peptides had at least one 9-mer frame predicted to bind to at least three SLA class II alleles; 80% (16 of 20 peptides) had at least one 9-mer predicted to bind to all four SLA class II alleles. From the external proteins, five of the predicted peptides were >85% identical in at least three IAV strains. Class II peptides derived from internal proteins were >85% identical in all seven IAVs, with exception of M_10 that had 84.2% identity (differed by 3 amino acids) with its counterpart in OH/04. Taken altogether, the immunoinformatics-predicted sequences represent a set of potentially broadly reactive swine influenza T cell epitopes.

### Epitope-driven DNA vaccine construction

As a tool to evaluate epitope-specific responses to predicted peptides, we designed two prototype DNA vaccines; one containing class I-restricted epitopes and one containing class II-restricted epitopes (S1 Text). To minimize potential junctional immunogenicity of the class I construct, spacers (‘AAY’) were inserted at seven of 27 peptide junctions. In one case, a ‘breaker’ (‘GPGPG’) was introduced into the class II construct to disrupt the formation of junctional epitopes. Both constructs were designed to avoid potential transmembrane domains. The DNA vaccine vectors also contained signal sequences to target the string of epitopes to the proteasome or the secretory pathway. These signal sequences, UbA76 for class I and TPA for class II, were of human origin; however, BLAST analysis showed that amino acid sequences from both were 99% and 71% identical, respectively, to their swine counterparts.

### T cell immunogenicity

**Fig 4:**
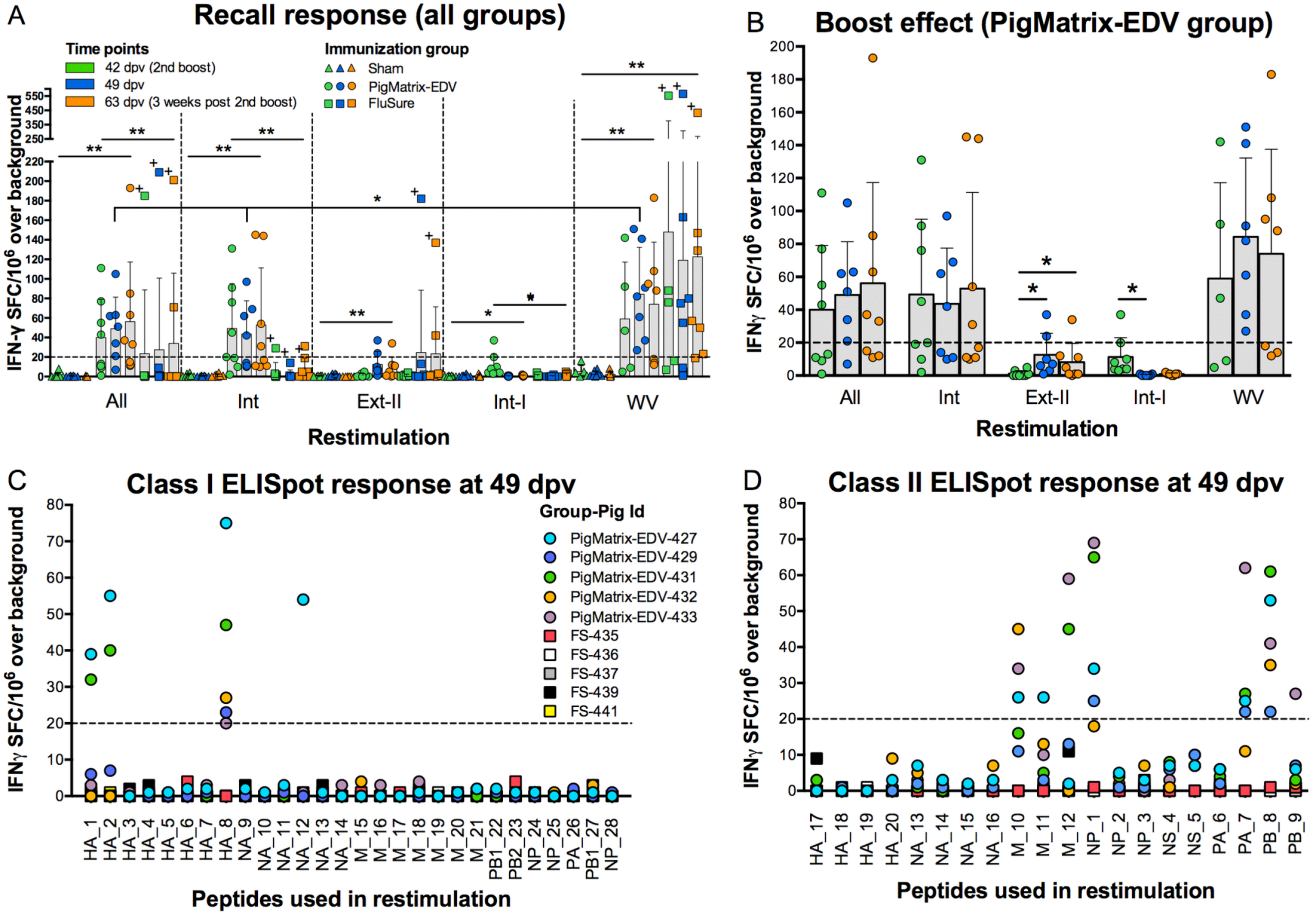
Peptide immunogenicity measured by IFNγ ELISpot. (A) PBMC (2.5 x 10^5^) isolated at three different time points (42, 49, and 63 dpv) from pigs immunized with empty plasmid (Sham), epitope-driven DNA vaccine (PigMatrix-EDV) and commercial vaccine (FluSure) were restimulated with pooled peptides (All, Int, Ext-II, and Int-I) at 10 μg/mL and whole virus (WV). The number of epitope-specific IFNγ spot forming cells (SFC) induced by the pools were measured using ELISpot assays. “High responder pig” (FS-442) is marked with +. (B) To evaluate vaccine boost effect, IFNγ responses to pooled peptides were measured at three different time points. For A and B, SFC over background, adjusted to spots per 10^6^ of PBMC seeded, are represented with bars indicating means and error bars indicating standard deviation (SD). Pooled peptide responses showing statistical significance when compared to Sham are indicated: **p<0.01, *p<0.05. Significant statistical difference for PigMatrix-EDV between restimulations at 49 dpv is also shown. Same colors and shapes are used in both figures. (C) PBMC from pigs vaccinated with PigMatrix-EDV and FluSure were restimulated with individual class I peptides and (D) class II peptides one week after the second boost (49 dpv). For C and D, SFC over background per 10^6^ PBMC are shown. A response was considered positive if the number of spots was greater than or equal to 20 SFC over background per 10^6^ PBMCs (dashed line).

Epitope-specific responses to pooled PigMatrix-predicted peptides were demonstrated in immune recall IFNγ ELISpot assays using PBMC isolated at 42 (day of second boost), 49, and 63 dpv from animals in the PigMatrix-EDV and Sham groups (Fig 4A). The four peptide pools (All, Int, Ext-II, and Int-I; Fig 2) used for restimulation induced statistically significant different responses between pigs vaccinated with PigMatrix-EDV and Sham (p<0.05). IFNγ responses measured in pigs from PigMatrix-EDV and FluSure groups were significatively different (p<0.05), with exception of restimulation with pool Ext-II. No significant differences were observed between pigs vaccinated with FluSure and Sham. In PigMatrix-EDV-vaccinated pigs, we expected class II epitopes to dominate in the immune response to external proteins, and class I epitopes to dominate the immune response to the internal proteins. Contrary to our expectation, the number of IFNγ SFC induced by pools of class II peptides from external proteins (Ext-II) and class I peptides from internal proteins (Int-I) was below 20, which was significantly lower (p<0.001) than responses to all peptides pooled together (All) and peptides derived from internal proteins (Int). SFC were not statistically different between All and Int pools (p = 0.74). These results suggest that immune responses to class II predicted epitopes contained in internal IAV proteins dominate the PigMatrix-EDV-induced response.

IFNγ SFC induced by restimulation with WV in PBMC from pigs in the PigMatrix-EDV group were also statistically different from Sham (p<0.05) at all three measured time points. This result suggests that T cells raised against epitopes contained in the prototype DNA vaccine recognize epitopes presented in whole virus stimulation *in vitro*.

It is interesting to note that the IFNγ SFC induced by restimulation with All and Int pools were not statistically different from responses to WV in PBMC from the PigMatrix-EDV group, with the exception of responses at 49 dpv (All: 49 (32.34), Int: 43.57 (33.93), WV: 84.29 (47.92) SFC per 10^6^ PBMC; p = 0.02; Fig 4A). This may suggest that the epitopes in the peptide pools were recognized by T cells that were responsible for the majority of T cell responses to WV, *in vitro*. Differences in the antigen presentation processes (*in vitro*) for WV and peptides may explain the differences at 49 dpv [47]. Alternatively, WV RNA could have played a role in the expansion of T cell responses *in vitro* [48]. It is also possible that the IFNγ ELISpot assay only sampled a fraction of the antigen-specific cells present in the PBMC after *in vitro* stimulation; thus, technical limitations may explain comparable responses between pooled peptides and WV restimulation.

Interestingly, IFNγ responses to WV restimulation did not significantly differ in PigMatrix-EDV (73.84 (54.48)) and FluSure-immunized (127.55 (175.86)) pigs (Fig 4A). Note that the mean and high variability in the FluSure group was due to consistently high recall responses in PBMC from one “high responder pig” (FS-442; Fig 4A, marked with +) at the three time points tested (mean IFNγ SFC per 10^6^ PBMC excluding this pig was 58.52 (51)). Thus, the epitope-based vaccine elicited consistent IFNγ responses equivalent to those induced by a tetravalent commercial WIV vaccine.

Boost immunizations in the PigMatrix-EDV group did not result in significant changes in the number of IFNγ SFC when PBMCs were restimulated with All, Int pools and WV (Fig 4A and enhanced in Fig 4B).

#### Restimulation with individual peptides

As noted before, we suspected that recall response to peptide pools in PBMC from PigMatrix-EDV vaccinated pigs was primarily driven by class II predicted epitopes derived from internal IAV proteins. This observation was confirmed by evaluating IFNγ responses to individual peptides a week after the second boost (49 dpv) (Fig 4C and 4D). PBMC from pigs immunized with PigMatrix-EDV or FluSure (five from each group) were restimulated with individual class I and II peptides. Four class I peptides (derived from external proteins) and seven class II peptides (derived from internal proteins) induced more than 20 IFNγ SFC per 10^6^ PBMC over background for at least one pig immunized with PigMatrix-EDV. At 49 dpv, none of the peptides induced significant responses in PBMC from the five FluSure-vaccinated pigs tested. Note that the high responder pig from the FluSure group, who registered the highest responses to peptide pool restimulation at 42, 49, and 63 dpv (FS-442, Fig 4A, marked with +), was not included in the individual peptide restimulation assays.

### Retrospective analysis using cohort-specific predictive matrices

Putative epitopes were predicted for binding to a set of SLA alleles prevalent in outbred swine populations [33,34]. To determine if those alleles were expressed in the study cohort, SLA types were determined at low resolution [33,34] at the end of the study for eight pigs from the Sham group, seven from PigMatrix-EDV, seven from FluSure, and two from NV group (S2 Table). By chance, none of the SLA-typed pigs tested in individual peptide ELISpot assays (Table 1), expressed any of the alleles used for epitope predictions. A pig that responded to four class I peptides (PigMatrix-EDV-427) was not SLA-typed; thus, no correlation of immune recall and epitope predictions could be made between the existing matrices and these ELISpot data.

**Table 1:** Low resolution SLA-type alleles of pigs tested in individual peptide ELISpot assays.

		SLA class I^a^		SLA class II^a^
Group	Pig	SLA-1	SLA-2	DRB1
PigMatrix-EDV	429	08XX,12XX,1301	0901–02,12XX	06XX,10XX
	431	08XX,12XX,1301	05XX,10XX	06XX,10XX
	432	08XX	05XX,10XX	10XX
	433	08XX,12XX,1301	0901–02,12XX	06XX,10XX
FS	435	1103,12XX,1301	10XX,jh02	06XX
	436	08XX	05XX,12XX	0401–02,10XX
	437	12XX,1301	10XX	06XX
	439	08XX	12XX	0401–02
	441	1103,12XX,1301	10XX,jh02	06XX

^a^Only loci for which prediction matrices were developed are shown.

To retrospectively evaluate the IFNγ responses to individual peptides and the association with specific SLA haplotypes, we developed class I and II matrices specific for the most frequent SLA-1, SLA-2, and SLA-DRB1 alleles expressed in the actual cohort (cohort-specific, Table 1). Although certain low-resolution results were ambiguous, we can make some assumptions based on common associations. For SLA class II, based on common association with DQB1 and DQA alleles [34], we expect that SLA-DRB1*0401–02 is likely to be DRB1*0402 and DRB1*06XX is likely to be DRB1*0602. These two alleles were expressed in 79% of the typed pigs. For the rest of the frequently expressed alleles, we developed XX01 as the default matrix (e.g. for DRB1*07XX, we developed SLA-DRB1*0701 prediction matrix). Thus, we developed cohort-specific prediction matrices for SLA-1*0801, 1*1201, 1*1301, 2*0501, 2*1201, DRB1*0402, 0602, 0701, and 1001.

**Fig 5:**
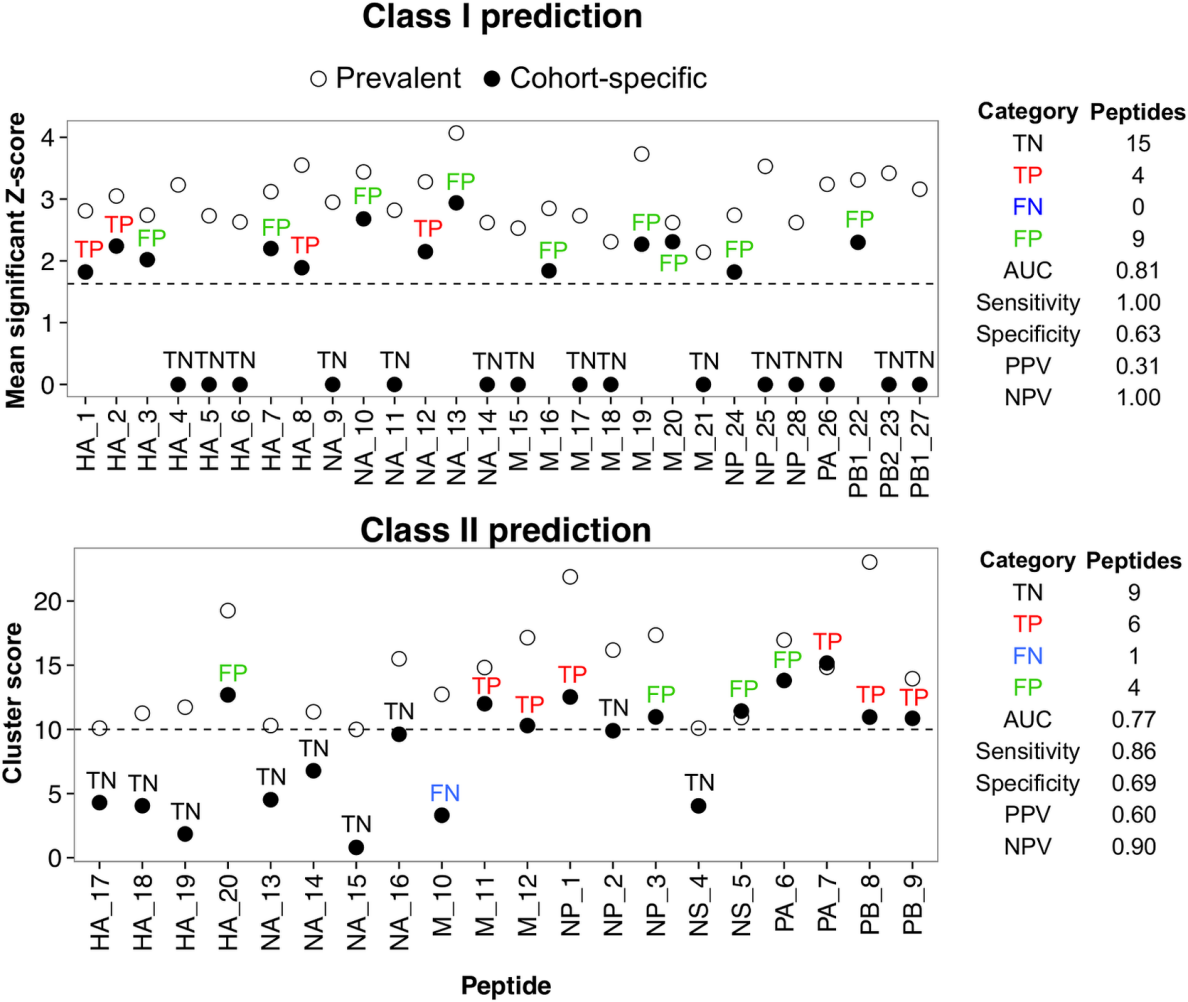
Comparison between prediction for prevalent and cohort-specific SLA alleles. Peptides were predicted to bind to a set of previously reported class I and class II SLA alleles prevalent in the U.S. swine population (prevalent). Based on low-resolution SLA-typing results, those alleles were not represented in the studied pigs. Prediction matrices were developed to predict binding potential of peptides to the most frequent SLA alleles found in the cohort (cohort-specific). (Top) Mean of significant Z-scores (above 1.64) over prevalent class I SLA alleles (SLA-1*0101, 1*0401, 2*0101, and 2*0401) and cohort-specific (SLA-1*0801, 1*1201, 1*1301, 2*0501, and 2*1201) are shown for each peptide. Peptides with a mean of significant Z-scores above 1.64 (dashed line) are considered potential binders. (Bottom) Cluster scores calculated for prevalent class II SLA alleles (DRB1*0101, 0201, 0401, and 0601) and cohort-specific alleles (DRB1*0402, 0602, 0701, and 1001) are shown for each peptide. Cluster scores above 10 (dashed line) are considered as potential binders. Based on the retrospective evaluation, peptides were classified in four categories (TN: true negatives, TP: true positives, FN: false negatives, and FP: false positives). AUC, Sensitivity, specificity, positive predictive value (PPV) and negative predictive value (NPV) are shown.

The initial set of peptides was selected because they were predicted to bind promiscuously to the SLA alleles that are prevalent in outbred swine populations (SLA-1*0101, 1*0401, 2*0101, 2*0401, DRB1*0101, 0201, 0401, and 0601). However, a reduced number of peptides were predicted to bind to the actual, cohort-specific alleles, once this information was available (Fig 5). For example, none of the peptides were predicted to bind the most frequently expressed SLA allele for this cohort (SLA-1*0801). Cohort-specific predictions yielded a total number of hits per allele, for this set of peptides, that was 41.7% lower than the initial prediction based on reported prevalent alleles. Despite the fact that the predictions did not correspond well with the sampled SLA, 23 PigMatrix-EDV peptides were still predicted to bind to alleles in the cohort, explaining the responses observed in the pool restimulation. This also suggests that initial predicted promiscuity (i.e. ability for a peptide to bind to multiple alleles) present in selected peptides extends to additional cohort-specific alleles.

#### Cohort-specific class I predictions

Further evaluation of the cohort-specific predictions revealed that PigMatrix predictions for the 28 class I peptides (Fig 5, top) had high sensitivity (1.0) and NPV (1.0), and moderate specificity (0.63). Cohort-specific prediction correctly classified 15 of the 24 peptides that were non-immunogenic. In terms of immunogenic peptides, four out of four were predicted as immunogenic, though nine false positive peptides were also observed, contributing to the low PPV (0.31).

To evaluate the predictive performance of the cohort-specific prediction, we built an ROC curve and then calculated the AUC (a value of 1 corresponds to a perfect prediction and 0.5 to a random prediction). The AUC was 0.81, which shows that predictions for cohort-specific alleles had high predictive power. However, the size of the dataset may have influenced these results; prospective studies on larger cohorts of pigs would be required to validate this observation.

#### Cohort-specific class II predictions

Predictions targeting cohort-specific SLA alleles showed that peptides had limited binding likelihood. Cohort-specific cluster scores were lower for 18 out of 20 peptides compared to cluster scores for SLA alleles reported as prevalent in outbred pigs (SLA-DRB1*0101, 0201, 0401, 0601; Fig 5, bottom). Note that the cohort-specific cluster score of false negative peptide M_10, which calculated for four SLA-DRB1 alleles (DRB1*0402, 0602, 0701, and 1001), was below 10 (the threshold we set for potential binders), but it was still predicted to bind to the two SLA-DRB1 alleles (DRB1*0602 and 1001) expressed by PigMatrix-EDV SLA-typed pigs (pig PigMatrix-EDV-432 only expressed SLA-DRB1*1001), and corresponded to the positive responses observed.

Cohort-specific class II prediction (Fig 5, bottom) had high sensitivity (0.86) and NPV (0.90), and moderate specificity (0.69) and PPV (0.60). They also showed high predictive power (AUC 0.77) for the set of 20 class II peptides. Nine of the 13 peptides that were non-immunogenic were correctly predicted and six out of seven immunogenic peptides were predicted as immunogenic. Only four peptides that were predicted to be immunogenic were non-immunogenic in T cell assays.

Overall, the retrospective analysis of 48 peptides using predictions for cohort-specific SLA alleles showed high sensitivity, moderate-to-high specificity and high predictive power for both class I and II SLA alleles. Predictions were particularly effective identifying non-immunogenic peptides as demonstrated by their high NPVs. Cohort-specific predictions correctly identified 24 out 37 non-immunogenic peptides and 10 out of 11 positive peptides. Still, it is important to mention that the limited number of peptides makes these results less robust. A larger dataset of peptides will be required to confirm the predictive power of the matrices.

### Antibody responses

FluSureXP^®^ induced detectable HI antibody against OH/07 γ-cluster virus, with no cross-reactivity to the H1N1pdm09 virus with serum collected 42 dpv (Table 2). HI antibody titers against OH/07 were significantly different from Sham and PigMatrix-EDV (p<0.001). PigMatrix-EDV did not induce a detectable positive HI antibody response against H1N1pdm09 or OH/07 at 42 dpv, which is not surprising, as the T cell epitopes were not expected to encode neutralizing B cell epitopes.

**Table 2:** Geometric mean reciprocal titers of HI antibodies to different virus in sera collected at 42 dpv.

	Viral antigen*	
Vaccine group	H1pdm09	OH/07
NV	7	6
Sham	6	6
PigMatrix-EDV	10	6
FluSure	8	104

*Titers <40 are considered negative or suspect.

## Discussion

In this study, PigMatrix, an immunoinformatics tool for predicting swine T cell epitopes, was used to identify class I and II epitopes highly conserved among seven IAV strains representative of those prevalent in U.S. swine. To evaluate the immunogenic potential of the predicted peptides, IFNγ SFC recall responses were measured in pigs vaccinated intramuscularly with prototype DNA vaccines (PigMatrix-EDV) encoding strings of class I and II epitopes or a commercially available swine IAV vaccine. Recall responses induced by pooled peptides in PBMCs isolated from pigs vaccinated with PigMatrix-EDV were significantly greater than responses in pigs vaccinated with empty plasmids. Furthermore, PigMatrix-EDV-vaccinated pigs responded to WV (H1N1pdm09) restimulation, showing that the epitope-based immunization gave rise to T cells that are cross-reactive with epitopes present in the whole virus *in vitro*. In addition, overall responses to WV restimulation were comparable to those induced by All and Int pools. Moreover, epitope-specific recall responses to WV in pigs immunized with a prototype epitope-based vaccine were similar to responses in pigs immunized with the commercial vaccine.

A challenge study was conducted to evaluate protective efficacy of PigMatrix-EDV. Pigs were intranasally challenged with H1N1pdm09 virus, but due to age at challenge and route of challenge, pathology and viral load in non-vaccinates was limited, so assessing protection overall in vaccinates was also limited. There was no evidence of enhanced lesions (VAERD) in vaccinates, and outcome in DNA-vaccinates and FluSure vaccinates was similar (data not shown). Future work aimed at assessing efficacy of the DNA approach is warranted, and further consideration will need to be given to animal age, route of challenge, challenge strain, and SLA haplotype of animals to adequately evaluate the vaccine. Ideally, a group of pigs challenged with influenza A virus should be included to evaluate whether the predicted T cell epitopes are also induced during natural infection.

Our initial set of alleles used for T cell epitope prediction did not correspond well with the cohort ultimately selected. For this prospective study, we developed predictions for SLA alleles that had been reported to be frequently expressed in outbred swine populations [33,34]. However, post hoc SLA typing results showed that those alleles were not prevalent in pigs in the study. Still, some peptides induced IFNγ SFC responses, demonstrating the initial set of alleles positively predicted promiscuous epitopes. This has significant implications for vaccine design because identification of epitopes capable of binding to multiple SLA alleles limits the number of epitopes required to cover an SLA diverse population. A retrospective analysis using cohort-specific alleles showed that some of the peptides were predicted to bind to the new SLA alleles, although the set of peptides overall was not optimally matched to the cohort. These results indicate that selecting epitopes for promiscuity, when pig SLA-typing is not available, may be relevant because conservation of binding likelihood in a promiscuous epitope may extend to additional (untested) alleles. While it is clear that we will need to expand the set of alleles for future vaccine designs, this finding suggests that using immunoinformatics tools to identify promiscuous T cell epitopes can contribute to those future designs [28,49].

Designing epitope-based vaccines for pigs is hindered by the lack of information on SLA diversity in the U.S. swine population. A systematic evaluation of the SLA frequency will make it possible to develop and apply predictions for the most representative SLA alleles (supertypes) [50,51] to vaccine designs that cover a high percentage of the swine population. In addition, a more streamlined (i.e. rapid, high resolution, commercially available) approach to SLA typing would significantly improve the ability to study T cell responses to influenza and other economically important diseases such as porcine reproductive and respiratory syndrome (PRRS) and porcine epidemic diarrhea (PED).

In this study, class II peptides from internal proteins were highly conserved (identity >85%) across all the analyzed strains and were shown to be the most immunogenic. Internal proteins from IAV are conserved across multiple strains because of the prevalence of two evolutionary lineages, H1pdm09 and TRIG, in the U.S. swine population [35]. We note that the genome sequences of the strains in the commercial vaccine are not available; however, it is likely that the internal epitopes were from the TRIG cassette (all seed strains predate introduction of H1N1pdm09 into the swine population). For this reason, it was interesting to see that PBMC from pigs immunized with FluSure had more limited IFNγ SFC responses to peptide pools, even though the pigs expressed similar SLA alleles to pigs in the PigMatrix-EDV group. This observation supports the hypothesis that epitope-based vaccines promote more efficient processing and presentation of their own epitopes as compared to whole-protein-based vaccines. Similar results were observed in mouse studies using T cell epitope-based DNA vaccines for *H*. *pylori*, where 33 out of 50 peptides stimulated more than 50 IFNγ SFC in splenocytes from the group vaccinated with a epitope-based DNA vaccine, but only two of the peptides were recognized in the group vaccinated with the whole bacteria lysate [52]. If epitope-based vaccines are able to induce immune responses to more individual epitopes than whole pathogen formulations containing the same epitopes, selection of the right epitopes, with the right breadth of SLA coverage, may lead to the development of more efficacious vaccines than currently exist [52].

Contrary to our expectations, we observed that IFNγ recall responses to class I peptides were restricted to *external* proteins, while responses to class II peptides were focused on epitopes derived from *internal* proteins. In human studies, most cross-reactive CD8+ (class I) and CD4+ (class II) T cell epitopes are derived from internal IAV proteins [14,53]. Compared to class I epitopes derived from internal proteins, HA- and NA-specific class I epitopes are said to be rare [54], but a few SLA-restricted HA and NA class I peptides have been reported [38,55]. In this study, the four class I peptides that induced IFNγ responses were derived from HA and NA. Sequence alignments using BLAST demonstrated that these peptides are conserved in different swine IAV strains. In humans, class II epitopes derived from HA and NA have also been reported [14,24,56], but none of the potential epitopes from these antigens predicted by PigMatrix elicited measurable responses in the PigMatrix-EDV group (Fig 4D). The seven class II peptides recognized by PBMC in this study were derived from internal proteins (M, NP, PA, and PB). Similar to class I peptides, these peptides are conserved in IAVs. Previous studies have shown that cross-reactive T cell responses to conserved epitopes may provide broader protection against diverse strains than antibodies that target variable antigens [15,24].

We searched the Immune Epitope Database (www.iedb.org) for swine influenza T cell epitopes and found that substrings of the predicted class II peptides NP_1, PA_7, PB_8, and M_12 have been reported to induce positive T cell responses, as measured by different methods (e.g. IFNγ ELISpot, tetramer staining, intracellular cytokine staining), for at least one human MHC class II allele. The published epitopes were derived from H1N1, H5N1, and H2N2 IAV strains. Thus epitopes that induce T cell responses in both human and pigs can be identified. Additionally, these epitopes may contribute to heterosubtypic cell-mediated responses against zoonotic IAV.

We did not expect that the epitope-based vaccine would induce antibodies, and indeed, PigMatrix-EDV did not induce HI antibodies that reacted to OH/07 or H1N1pdm09. While the commercial vaccine induced antibodies against OH/07, they did not cross-react with H1N1pdm09. The commercial vaccine contains four IAV strains (H1γ, H1δ, H1δ, H1N1 viruses, and one cluster IV H3N2 virus). OH/07 is an H1γ virus, which explains the positive HI response to this virus.

In conclusion, observed epitope-specific IFNγ recall responses demonstrate the potential for PigMatrix to predict conserved, promiscuous and immunogenic T cell epitopes. Further studies will evaluate the utility of PigMatrix for designing epitope-driven vaccines for swine. Epitope-driven T cell responses may not fully prevent IAV infection, but could reduce viral burden, as was observed for the 2009 H1N1 outbreak [24]. Rapid viral clearance and lower morbidity are important objectives for swine IAV vaccines, since current vaccines do not provide complete protection against variant strains. Moreover, epitope prediction tools could be used to assess the potential for existing commercial vaccine strains to protect against newly emergent strains of IAV. Improved immunoinformatics tools that target a comprehensive set of SLA alleles may contribute to the development of vaccines against other prominent swine diseases and provide a significant positive impact for pig health and swine producers.

## Supporting Information

S1 Table GenBank identification numbers of gene sequences of proteins expressed by representative swine IAV. (PDF)

S2 Table Low-resolution SLA-typing results. (PDF)

S1 Text Concatemer construct sequences. (PDF)
