# Supplementary material for: In Vivo Validation of Predicted and Conserved T Cell Epitopes in a Swine Influenza Model
Source: PLoS One. 2016 Jul 13;11(7):e0159237. doi: 10.1371/journal.pone.0159237 (PMC4943726; doi:10.1371/journal.pone.0159237)
Supplement: S1 Table — (PDF) [file pone.0159237.s001.pdf]

S1 Table. GenBank identification numbers of gene sequences of proteins expressed by representative swine IAV

	GenBank id							
Strain	PB2	PB1, PB1-F2	PA	HA	NP	NA	M2, M1	NS2, NS1
A/California/04/2009(H1N1)	227809823	332384205	227977113	229535948	332384201	332384199	332384196	332384209
A/swine/Illinois/5265/2010(H1N1)				290873719		290873721	290873723	
A/swine/Ohio/511445/2007(H1N1)	197344175	197344177	197344180	197344171	197344182	197344173	197344184	197344187
A/swine/Minnesota/02011/2008(H1N2)	304272397	304272373	304272351	304272327	304272303	304272279	304272249	304272213
A/swine/Minnesota/A01301731/2012(H1N2)	421920014	421920016	421920018	421920020	421920022	421920024	421920026	421920029
A/swine/Texas/4199-2/1998(H3N2)	340784749	340784753	340784756	340784760	340784762	340784766	340784768	340784771
A/turkey/Ohio/313053/2004(H3N2)	361630452	367462658	367462657	371574609	367462656	370321100	361630450	361630451
