# Supplementary material for: In Vivo Validation of Predicted and Conserved T Cell Epitopes in a Swine Influenza Model
Source: PLoS One. 2016 Jul 13;11(7):e0159237. doi: 10.1371/journal.pone.0159237 (PMC4943726; doi:10.1371/journal.pone.0159237)
Supplement: S2 Table — (PDF) [file pone.0159237.s002.pdf]

S2 Table. Low-resolution SLA-typing results

		SLA class I			SLA class II		
Group	Pig	SLA-1	SLA-2	SLA-3	DRB1	DQB1	DQA
NV	413	08XX	0901-02	05XX,0602	07XX,10XX	0201,06XX	01XX,02XX
	414	08XX,15XX	12XX,16XX	0601,07XX	0401-02,0403-04	0202,0302-03	02XX
Sham	419	08XX,15XX	05XX,16XX	07XX	0403-04,10XX	06XX,07XX	01XX
	420	08XX	0901-02,12XX	05XX,0601	0401-02,07XX	0201,0202	02XX
	421	08XX,12XX,1301	05XX,10XX	05XX,07XX	06XX,10XX	06XX,07XX	01XX
	422	08XX,15XX	12XX,16XX	0601,07XX	0401-02,0403-04	0202,0302-03	02XX
	423	08XX	0901-02,12XX	05XX,0601	0401-02,07XX	0201,0202	02XX
	424	08XX	12XX	0601	0401-02	0202	02XX
	425	12XX,1301	10XX	05XX	06XX,10XX	06XX,07XX	01XX
	426	08XX	05XX,12XX	0601,07XX	0401-02,10XX	0202,06XX	01XX,02XX
PigMatrix-EDV	428	08XX	05XX,12XX	0601,07XX	0401-02,10XX	0202,06XX	01XX,02XX
	429	08XX,12XX,1301	0901-02,12XX	05XX,0602	06XX,10XX	06XX,07XX	01XX
	430	08XX	05XX,12XX	0601,07XX	0401-02,10XX	0202,06XX	01XX,02XX
	431	08XX,12XX,1301	05XX,10XX	05XX,07XX	06XX,10XX	06XX,07XX	01XX
	432	08XX	05XX,10XX	05XX,07XX	10XX	06XX	01XX
	433	08XX,12XX,1301	0901-02,12XX	05XX,0602	06XX,10XX	06XX,07XX	01XX
	434	04XX,08XX	04XX,12XX	04XX,0601	0401-02,09XX	0202,08XX	02XX,03XX
FluSure	435	1103,12XX,1301	10XX,jh02	05XX	06XX	07XX	01XX
	436	08XX	05XX,12XX	05XX,0601	0401-02,10XX	0202,06XX	01XX,02XX
	437	12XX,1301	10XX	05XX	06XX	07XX	01XX
	438	08XX,12XX,1301	05XX,12XX	05XX,0601	0401-02,06XX	0202,07XX	01XX,02XX
	439	08XX	12XX	0601	0401-02	0202	02XX
	440	07XX,08XX	02XX,05XX	04XX,07XX	02XX,10XX	0201,06XX	01XX,02XX
	441	1103,12XX,1301	10XX,jh02	05XX	06XX	07XX	01XX
