# Supplementary material for: In Vivo Validation of Predicted and Conserved T Cell Epitopes in a Swine Influenza Model
Source: PLoS One. 2016 Jul 13;11(7):e0159237. doi: 10.1371/journal.pone.0159237 (PMC4943726; doi:10.1371/journal.pone.0159237)
Supplement: S1 Text — (PDF) [file pone.0159237.s003.pdf]

S1 Text. Concatemer construct sequences

```
>Class I concatemer construct
cggatctaccagattctggccatctacatcaccatcggcaagtgccccaagtactctctgc
tgaccgaagtggaaacatatggccccggacccggcaccagtgccgatcagcagtctctgta
cggacccggacccggggagatgaacgcccccaactatcactatctggccagctgcatggga
ctgatctacgacaccgtgaaccggacccaccagtacggcaccgagaagctgaccatcacat
acgccttcgacgagcggcggaacaaatacgataccgtgcacgaccggacccccctatctgac
tgaagtggaaacttacgtgctgggacccggacccggcagcgtgaagaatggcacatacgac
tacggcaccatcaaggacagaagcccctacaacgccgacacactgtgcatcggctatgggc
ccggacccggggggaatggtggatgggtggtacggctactgcaccgagctgaagctgagcga
ctatggacccggacccggcgtgtcagatggcggcccaaatctgtacgaaatctgccccaag
ctggccgagtacaagagctgcatcaaccggtgcttctaccaagtgtcccggcccatgtttc
tgtacggcgccaaagaagtggctctgagctacggacccggacccggcgaactggatgcccc
aaactaccactacgatctgctggaaaatctgcaagcctacgccagccaaggcaccaagcgg
agctacaacaccgatctggaagctctgatggaaaacatggacaaggccgtgaagctgtatc
tgagcaccgccagcagctggtcttacggacccggacccggcggaatgatcgacgggtggta
tggatactgatga

>Class II concatemer construct
acaagaggcgtgcagatcgccagcaacgagaacgtggaaaccatggacagcaacacactgg
aactgcggacatacgtgctgagcatcatccccagcggccctctgaaggccgagatcgccca
gagactggaaagcgtgtacagatacggcttcgtggccaacttcagcatggaactgcccagc
ttcggcgtgtccggctgcagaacattctttctgacacaaggcgctctgctgaacgacaagc
acatcgaccccttcaagctgctgcagaatagccaagtggtgtctctgatgcggccctacga
ggaactgcgcgagcagctgtccagcgtgtccagcttcgagcggttccaagacattctgatg
cgcatgagcaagatgcagctgggctccagcagcgagatgatgggcatgttcaatatgctga
gcaccgtgctgggcgtgtccatcggcgacaagatcacattcgaggccaccggcaatctggt
ggtgccccggtacagatccaagtttctgctgatggacgctctgaagctgtccatcgaggac
cctagctgcatgggactgatctacaaccggatgggcaccgtgaccaccgaggccgcttttg
gactcgtgtgcttcgagcagatcactttcatgcaagctctgcagctgctgctggaagtgga
aaaccagacttacgtgaacatcagcaacaccaacttcgccgctggccagagcgtggtgtcc
gtgaagctggtgcccagatacgcctttgccatggaacggaatgccggcagcggcatcatca
tcagcgaagtgcacatctactatctggaaaaggccaacaagatcaagagcgagaaaaccca
catccacatctttggccccggacccggcatggccaatctgattctgcagatcggcaacatc
atctccatctggatcagccactctatcgaggatctgatctttctggctcggagcgcactga
ttctgagaggcagcgtggcccacaagagctgtctgcccaccagacagatggtgcacgccat
gagaaccatcggcacccaccctagctctagcgcctccgtggtgtctgtgaaactggccggc
aatagctctctgtgccccgtgtccacccggatctaccagattctggccatctacagcaccg
tggcctcttctctggtgctcgtgtgatga
```
